# Impacts of the COVID‐19 pandemic on human–nature interactions: Pathways, evidence and implications

**DOI:** 10.1002/pan3.10201

**Published:** 2021-04-06

**Authors:** Masashi Soga, Maldwyn J. Evans, Daniel T. C. Cox, Kevin J. Gaston

**Affiliations:** ^1^ Graduate School of Agricultural and Life Sciences The University of Tokyo Tokyo Japan; ^2^ Fenner School of Environment and Society The Australian National University Canberra ACT Australia; ^3^ Environment and Sustainability Institute University of Exeter Penryn UK

**Keywords:** behaviour, disease, distribution, extinction of experience, global change, personalised ecology

## Abstract

1. The coronavirus (COVID‐19) pandemic and the global response have dramatically changed people's lifestyles in much of the world. These major changes, as well as the associated changes in impacts on the environment, can alter the dynamics of the direct interactions between humans and nature (hereafter human–nature interactions) far beyond those concerned with animals as sources of novel human coronavirus infections. There may be a variety of consequences for both people and nature.
2. Here, we suggest a conceptual framework for understanding how the COVID‐19 pandemic might affect the dynamics of human–nature interactions. This highlights three different, but not mutually exclusive, pathways: changes in (a) *opportunity*, (b) *capability* and (c) *motivation*.
3. Through this framework, we also suggest that there are several feedback loops by which changes in human–nature interactions induced by the COVID‐19 pandemic can lead to further changes in these interactions such that the impacts of the pandemic could persist over the long term, including after it has ended.
4. The COVID‐19 pandemic, which has had the most tragic consequences, can also be viewed as a ‘global natural experiment’ in human–nature interactions that can provide unprecedented mechanistic insights into the complex processes and dynamics of these interactions and into possible strategies to manage them to best effect.

A free Plain Language Summary can be found within the Supporting Information of this article.

## Abstract 2

A free Plain Language Summary can be found within the Supporting Information of this article.

## 要約

1. 新型コロナウイルス感染症の流行（以降、「コロナ感染症」）や世界規模での感染症対策の実施は、人々のライフスタイルを劇的に変化させた。こうしたライフスタイルの変化やそれに伴う環境への影響の変化は、人間と自然との直接的な関わり合い（以降、「人と自然との相互作用」）のダイナミクスを、人畜共通感染症の文脈をはるかに超えた広い範囲で変化させる可能性がある。こうした人と自然の相互作用の変化は、人間と自然の双方に様々な影響をもたらすだろう。。
2. 本論文では、コロナ感染症が人と自然の相互作用のダイナミクスにどのような影響を与えるのかを理解するために有効な概念的枠組みを提案する。この枠組みは、人と自然の相互作用の変化が、（一）「機会」の変化、（二）「能力」の変化、（三）「動機」の変化という三つの異なる経路によってもたらされることを示している。
3. またこの枠組みは、コロナ感染症によって引き起こされた人と自然の相互作用の変化が、複数のフィードバックを介して新たな相互作用の変化をもたらし、その影響はコロナ感染症が収束した後も長期にわたって持続し得ることも示唆している。
4. コロナ感染症は極めて悲劇的な状況をもたらしたが、一方でこうした状況は人と自然の相互作用に関する「地球規模の自然実験」とみなすこともできる。この自然実験を利用することによって、人と自然の相互作用の複雑なプロセスとダイナミクスに関する新たな知見を獲得するとともに、この相互作用の適切な管理戦略を検討することができるだろう。

## 1 INTRODUCTION

**FIGURE 1:**
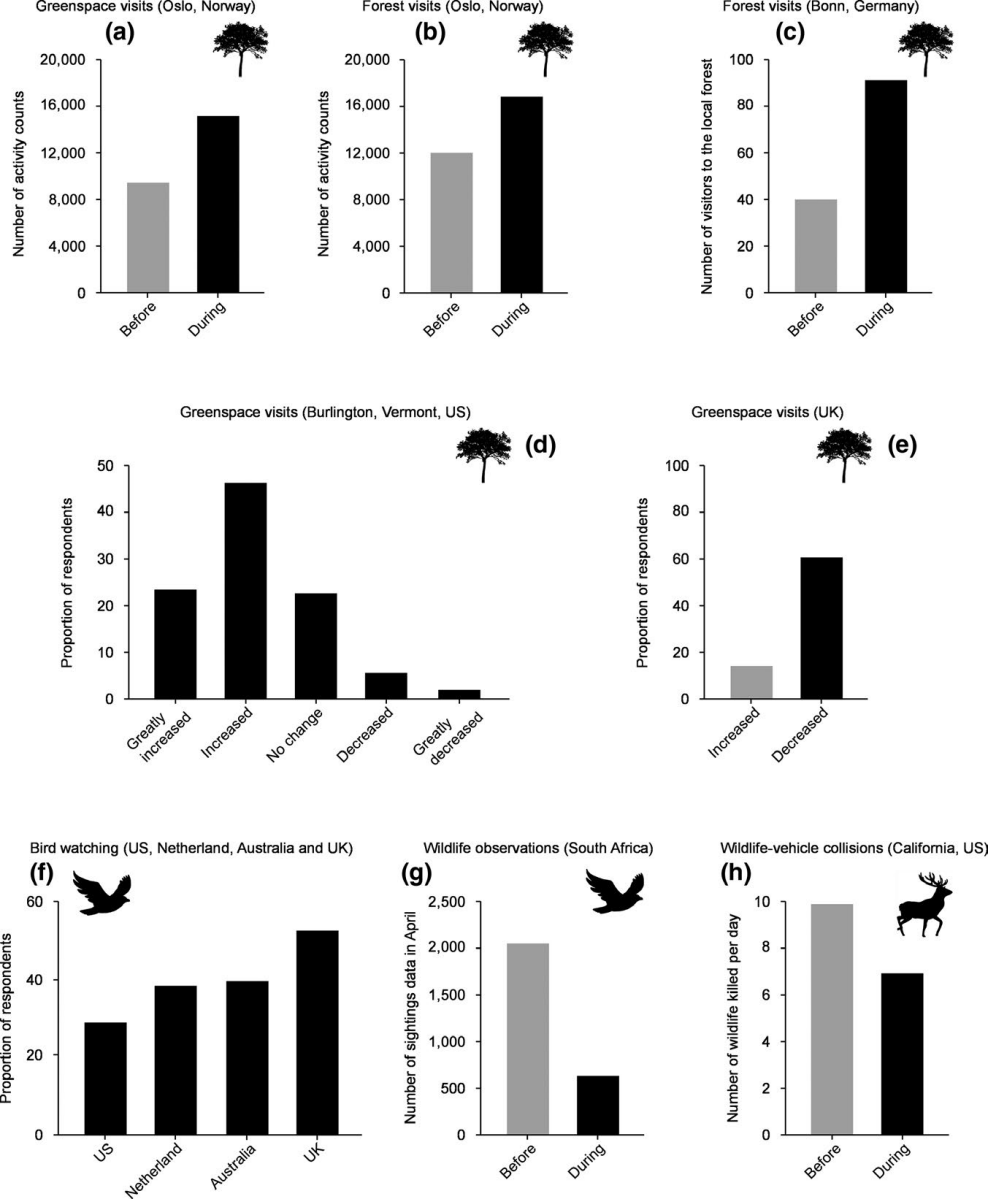
Empirical evidence suggesting changes in human‐nature interactions due to the pandemic. (a–b) Number of activity counts of people (measured by mobile tracking data) recorded in forests and urban greenspace before and during the COVID‐19 lockdown in Oslo, Norway (a: forests, b: urban greenspace; Venter, Aunan, et al., 2020; Venter, Barton, et al., 2020); (c) Number of visitors to the local forest before and during COVID‐19 lockdown in Bonn, Germany (note: we used data at 1 pm on Sunday; Derks et al., 2020); (d) Perceived changes in frequency of visits to natural environments during the pandemic compared to prior to it in Burlington, Vermont, US (Grima et al., 2020); (e) Proportion of respondents who reported an increase (grey) or decrease (black) in time spent in greenspace during lockdown compared to same period last year in the UK (Olsen & Mitchell, 2020); (f) Percentage of people (birders) reporting that the pandemic has shifted their birding behaviour to being more local (i.e. they have focused on their nearer environments and birding hotspots closer to their home) in four countries (Randler et al., 2020); (g) Number of sightings data submitted by citizen scientists (Southern African Bird Atlas Project) in April before (2019) and during the COVID‐19 lockdown in South Africa (Rose et al., 2020); and (h) Number of large bodied wild animals killed per day on California state highways before and after implementation of stay‐at‐home orders (Shilling et al., 2021)

The coronavirus (COVID‐19) pandemic (hereafter pandemic) and the resultant global response have dramatically altered people's daily lives. To curb the transmission of the virus, governments in many countries, regions and localities have implemented strict policies of social distancing and shelter‐in‐place (i.e. working from home or just staying home). These policies, together with more voluntary behavioural changes, have resulted in unprecedented shifts in human activity in a very short period, such as reduced travel, the closing down of much business activity and an increased time spent at home. At least in many developed countries, these rapid and widespread changes in human lives, along with the associated changes in anthropogenic pressures on the environment, have altered the dynamics of direct interactions between humans and nature (hereafter human–nature interactions). These changes have manifested in a variety of ways (Figure 1; e.g. Derks et al., 2020; Grima et al., 2020; Randler et al., 2020; Rose et al., 2020; Ugolini et al., 2020; Venter, Aunan, et al., 2020; Venter, Barton, et al., 2020; Shilling et al., 2021), and inevitably there has been a multitude of positive and negative consequences for both humans and nature. The pandemic is a new and sudden phenomenon, and as a result there has as of yet been limited scientific consideration of these effects. This article aims to stimulate such attention.

In this perspective, we examine the potential impacts of the pandemic on direct human–nature interactions. We consider a wide diversity of human–nature interactions, such as visiting a protected area or urban greenspace, viewing trees through a window, listening to bird song or being attacked by a bear. Following previous studies (Gaston et al., 2018; Soga & Gaston, 2020), however, we exclude ‘interactions’ with organisms that are not self‐sustaining (e.g. seeing potted houseplants, playing with domestic pets) and those through the media (e.g. viewing nature documentaries), although we acknowledge that, during this extraordinary period, such activities have been of importance to many people (Pérez‐Urrestarazu et al., 2020; Young‐Mason, 2020). In this piece, we also focus mainly on developed countries, albeit with reference to the global context. We describe a conceptual framework for understanding how the pandemic could affect the dynamics of human–nature interactions, discuss the potential consequences of the changes in these interactions and suggest key knowledge gaps and recommendations for further research (see Box 1). Finally, we go on to highlight that the pandemic constitutes an unintended (and undesirable) ‘global natural experiment’ (Thomson, 2020) in human–nature interactions that, without seeking to downplay or ignore its tragic consequences, provides a rare opportunity to produce in‐depth knowledge about these interactions and help establish novel actions and strategies that can have positive outcomes for both humans and nature.

BOX 1 **Examples of priority research questions regarding the impacts of the pandemic on human–nature interactions.**

Q1. How has the pandemic changed people's opportunity, motivation and capability to interact with nature?

Q2. What is the relative importance of opportunity, motivation and capability in changing people's levels of interactions with nature?

Q3. How are people's opportunity, motivation and capability to interact with nature related to each other?

Q4. How are different types of human–nature interactions affected by the pandemic?

Q5. How has the pandemic altered the composition of human–nature interactions?

Q6. How do the strength and directions of the impacts of the pandemic on human–nature interactions differ across populations, regions, countries and cultures?

Q7. Has the pandemic changed the importance of human–nature interactions for human health and well‐being?

Q8. How has the increased use of some natural environments (e.g. urban greenspace) during the pandemic influenced the ecological conditions of these environments?

Q9. How have the changes in the ecology of wildlife during the pandemic influenced human–wildlife interactions?

Q10. How has the prevalence of common mental disorders (e.g. depression) due to the pandemic influenced people's use of nature?

Q11. How has the increased fear and dislike towards bats influenced people's motivation to interact with nature?

Q12. What role can the pandemic play in limiting the extinction of experience?

Q13. How long will the impacts of the pandemic on human–nature interactions last?

## 2 CONCEPTUAL FRAMEWORK

**FIGURE 2:**
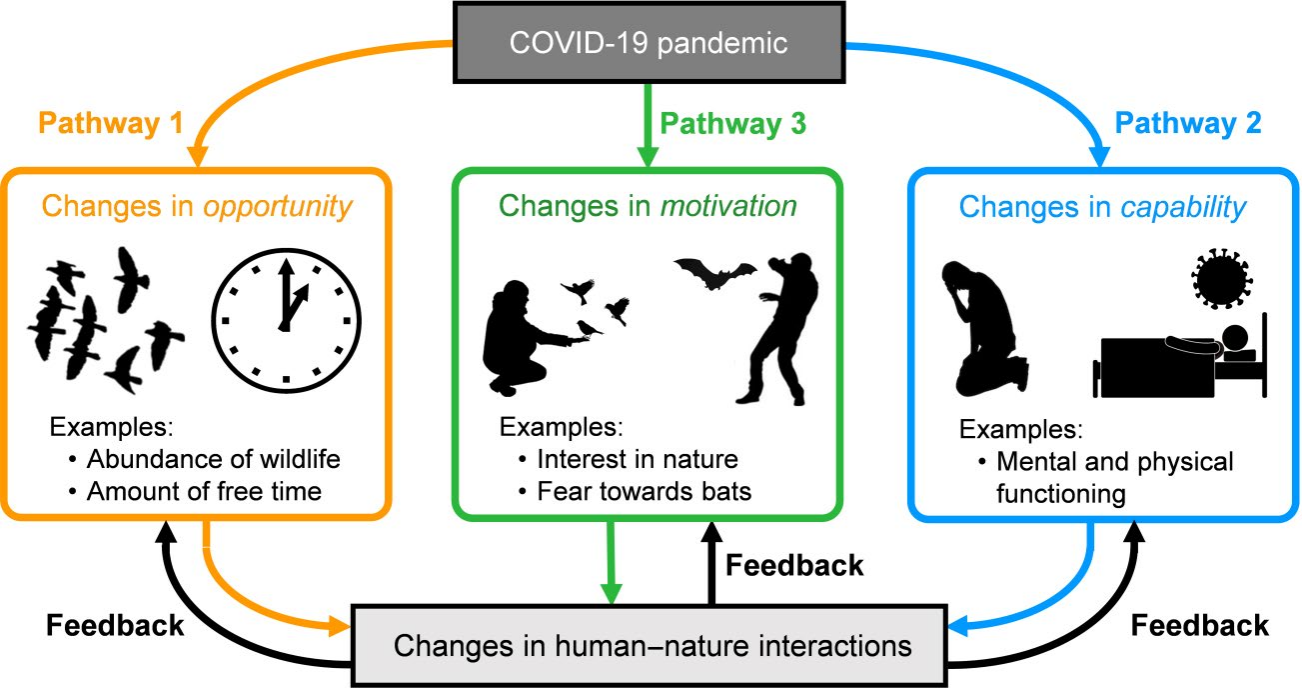
A conceptual framework for understanding how the pandemic could affect the dynamics of human–nature interactions. The pandemic could influence human–nature interactions through three major pathways: changes in *opportunity* (Pathway 1; orange arrows); changes in *capability* (Pathway 2; blue arrows) and changes in *motivation* (Pathway 3; green arrows). These three pathways are likely to be interrelated in various ways. There are likely also several feedback loops in which changes in human–nature interactions induced by the pandemic can lead to further changes in their dynamics (black arrows). Note that this schematic diagram does not necessarily represent all potential factors and processes

Adopting the COM‐B model of behaviour developed by Michie et al. (2011), we consider that the pandemic could influence the dynamics of direct human–nature interactions through three different, but not mutually exclusive, pathways: changes in *opportunity* (Pathway 1); changes in *capability* (Pathway 2) and changes in *motivation* (Pathway 3; Figure 2). The strength and direction of these three pathways likely vary substantially across populations, regions and countries due to socioeconomic, political, cultural and environmental factors as well as, potentially linked, variation in the severity and response to the pandemic (Box 1). In the following sections, we explain and justify this conceptual framework, and use it to explore how each of the three pathways contribute to changes in human–nature interactions as a result of the pandemic.

### 2.1 Pathway 1: Changes in *opportunity*

*Opportunity* concerns the factors that facilitate or make an interaction with nature possible. These include, for example, the amount of wildlife and natural environments that a person can interact with, the amount of time available for a person to spend engaging with nature, and social and cultural norms that affect a person's behaviour. The pandemic is likely to affect people's opportunity to interact with nature both positively and negatively (Table 1; Figure 2). On the positive side, for example, the adoption of remote working policies during the pandemic has increased some people's available time for other activities, which may promote their positive interactions with nature, such as visiting natural environments in their neighbourhood (Figure 1a–d; this increase in nature experiences maybe less relevant for those who are not able to engage in home working or remote working). Likewise, at least in developed regions and countries, during the pandemic many indoor amusement facilities, such as movie theatres, museums, bars and restaurants have been closed as a measure to reduce infection rates. This might be one of the major drivers of the increase in people's use of natural environments (e.g. urban parks) during the pandemic (Day, 2020; Derks et al., 2020; Grima et al., 2020; Kleinschroth & Kowarik, 2020; Venter, Aunan, et al., 2020; Venter, Barton, et al., 2020) because they often offer one of the only available alternatives for recreation or socialising. Increased use of neighbourhood natural environments is likely to contribute to improved human health and well‐being (Pouso et al., 2020; Soga et al., 2021), albeit it can, in some cases, have adverse ecological impacts on these environments (e.g. increased pressure on understorey vegetation; Box 1).

**TABLE 1:** Examples of possible changes in human–nature interactions due to the pandemic and their potential drivers and consequences. The three types of pathways (Pathways 1, 2 and 3) presented in the *Drivers* column correspond to those presented in Figure 2. Note that we just provide representative elementary examples, and the pandemic is likely to alter human–nature interactions in various ways

Possible changes	Drivers (Pathways)	Consequences
Increase in recreational use of natural environments	Increased interest in outdoor physical activity (*Pathway 3*); increased positive attitudes towards nature (*Pathway 3*) and increased availability of discretionary time (*Pathway 1*)	Improved health and well‐being in local human populations (e.g. decreased risk of lifestyle diseases) Increased pressures on wildlife species/decrease in wildlife abundance
Increase in frequency of bird feeding in domestic gardens	Increased positive attitudes towards nature (*Pathway 3*) and increased availability of discretionary time (*Pathway 1*)	Improved health and well‐being in local human populations (e.g. decreased risk of lifestyle diseases) Increased species richness and abundance of birds in urban and suburban areas
Increase in frequency of hearing bird song in urban areas	Increase in duration of singing in urban birds, and increased detectability of bird song due to decreased levels of background noise (*Pathway 1*)	Improved health and well‐being of human urban residents (e.g. decreased symptoms of depression)
Increase in number of human attacks by wildlife in suburban and rural areas	Increased abundance of problematic wildlife (e.g. bears) due to reduced human activity (*Pathway 1*)	Increased injury and death risks for people living in suburban and rural areas
Decrease in number of visitors to remote natural environments and ecotourism sites (e.g. national parks)	Reduced ability to travel due to travel restriction policies (*Pathway 1*) and poor physical and psychological health (*Pathway 2*); closure of national parks (*Pathway 1*)	Decrease in anthropogenic impacts on wildlife inhabiting national parks Reduced amount of citizen science data for threatened species Reduced number of wildlife attacks on humans
Decrease in number of wildlife–vehicle collisions	Reduced number of cars on highways due to reduced economic activity and human mobility (*Pathway 1*)	Reduced mortality in wildlife populations Reduced economic and social costs associated with collisions (e.g. car crash)

On the negative side, the spreading of social norms that prevent people from spending time outside (e.g. people should follow governmental stay‐at‐home orders) as well as increased fear of the virus during the pandemic are likely to discourage many from participating in outdoor activities, which would decrease their interactions with nature (Figure 1d). Loss of any interactions that would have occurred while travelling to work, and loss of free time because of additional responsibilities (e.g. home schooling, child and elderly care) can have similar effects. Likewise, the pandemic has made for an enormously busy period for some groups of people such as healthcare workers and delivery drivers, which will have reduced their available time for other activities including nature experiences. Furthermore, in some regions, urban parks, beaches and other recreational areas have, at least partly, been closed during the pandemic (Armstrong & Lucas, 2020), which has reduced people's opportunities directly to interact with nature.

The pandemic has likely changed not only the number of opportunities a person has to engage with nature, but also how they might interact with nature. For example, at least in urbanised regions, stay‐at‐home orders have made many people spend most of their time indoors (Greenwood‐Hickman et al., 2020; Moore et al., 2020). This shifts their opportunities towards ‘less immediate’ interactions with nature (sensu Soga & Gaston, 2020), such as viewing garden trees from a window or listening to outdoor bird song from inside a room. Likewise, the implementation of mobility restriction policies during the pandemic will have reduced people's opportunities to visit natural environments far from home (e.g. national parks; Randler et al., 2020; Rose et al., 2020; Figure 1f,g), although this might have instead increased their use of natural environments nearby (e.g. urban parks; Derks et al., 2020; Geng et al., 2021; Kleinschroth & Kowarik, 2020; Venter, Aunan, et al., 2020; Venter, Barton, et al., 2020). This change suggests that people's opportunities to have ‘less human‐mediated’ interactions with nature (i.e. those that can occur in places where it is little disturbed by humans) have been replaced with ‘more human‐mediated’ ones (i.e. those that can occur where anthropogenic influences are marked; Soga & Gaston, 2020). This, of course, depends on where people live. Those who reside in urban centres, for example, will have fewer opportunities for ‘less human‐mediated’ interactions than those who live in rural areas who might, on the other hand, have more opportunities to interact with nature while confined to the surrounds of their homes. Understanding the consequences of the changes in the composition of human–nature interactions is a key challenge that has received relatively little attention (Box 1).

**FIGURE 3:**
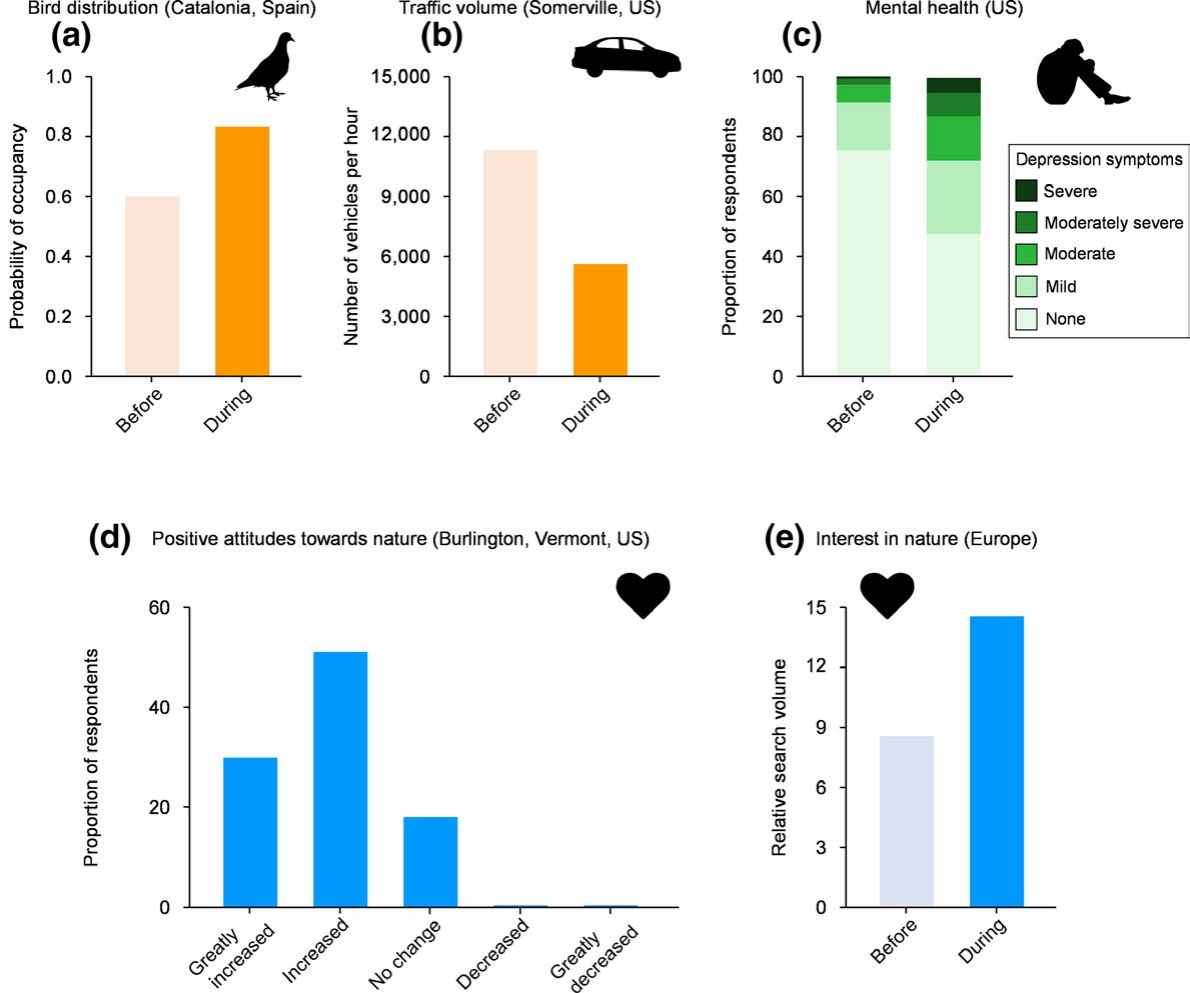
Empirical evidence suggesting the presence of the three pathways (a, b: changes in *opportunity*, c: changes in *capability*, d, e: changes in *motivation*) through which the pandemic affects human‐nature interactions (see also Figure 2). (a) Probability of occupancy of Rock pigeon *Columba livia* in urban areas before and during COVID‐19 lockdown in Catalonia, Spain (Derks et al. 2020); (b) Diurnal traffic volume (vehicles/h) on weekdays on urban roadways between March and May before (in 2018) and during the pandemic in Somerville, the U.S. (we used data at 1 pm; Hudda et al., 2020); (c) Prevalence of depression symptoms in US adults before and during the pandemic (depression symptoms were assessed using the Patient Health Questionnaire; Ettman et al., 2020); (d) Perceived changes in personal importance of being able to access natural environments during the pandemic compared to prior to it in Burlington, Vermont, US (Grima et al., 2020); and (e) Relative search volume for nature‐related topics (e.g. forest, bird, nature, biodiversity) on Google before and during the pandemic in 20 European countries (Rousseau & Deschacht, 2020)

The unprecedented reduction in global economic and transport activity due to the pandemic has dramatically reduced the impacts of anthropogenic disturbances on ecosystems worldwide (Diffenbaugh et al., 2020), the so‐called 'anthropause' (Rutz et al., 2020). Indeed, in many regions (especially in more developed societies), there have been noticeable decreases in anthropogenic pollution (e.g. air and water pollutants, noise, artificial light) due to reduced economic activity and human mobility (e.g. Bustamante‐Calabria et al., 2021; Chen et al., 2020; Derryberry et al., 2020; Mandal & Pal, 2020; Venter, Aunan, et al., 2020; Venter, Barton, et al., 2020), and in some there has been a reduction in management (e.g. cutting vegetation) associated with greenspaces (e.g. road verges, urban greenspaces; K.J.G., pers. obs.). Furthermore, shelter‐in‐place and stay‐at‐home orders and related actions (e.g. closure of non‐essential businesses) implemented during the pandemic have reduced the number of passenger cars on major roadways (Hudda et al., 2020; Figure 3b), which is likely to result in the reduction of collisions between vehicles and wildlife (Shilling et al., 2021; Figure 1h). These reduced anthropogenic impacts on ecosystems can modify the behaviour and distribution of some wildlife species relatively quickly (Rutz et al., 2020), which, in turn, affects the dynamics of human–nature interactions (Figure 2). For example, it is known that urban birds have altered their behavioural patterns (e.g. acoustic signalling, timing of singing) in response to reduced noise pollution during the pandemic (Derryberry et al., 2020; Gordo et al., 2020), which has changed (in many cased increased) people's frequency of hearing bird song (Gordo et al., 2020; Figure 3a). An increase in the frequency of hearing bird song in urban areas might have had favourable outcomes upon human health and well‐being; this type of nature interaction is known to be associated with improved psychological health (Ratcliffe et al., 2013; Table 1). Likewise, in developed regions and countries, there have been many anecdotal observations of wild animals venturing into urban and suburban areas where they have not been seen regularly or for many years, as a consequence of traffic and other human activity declining during the pandemic (c.f. Zellmer et al., 2020). The expansion of the distribution and abundance of problematic wildlife species (e.g. bears), as well as their behavioural changes (e.g. increased aggressive behaviours), can increase the frequency of negative human–nature interactions, such as being attacked by wildlife or disturbed by its activities (Table 1). Quantifying how behavioural and distributional changes in wildlife alter human–wildlife interactions will help determine appropriate actions and strategies for the management of these interactions (Box 1).

### 2.2 Pathway 2: Changes in *capability*

*Capability* is an individual's psychological and physical capacity to engage in interactions with nature. The pandemic is likely to change a person's capability to interact with nature (Table 1; Figure 2). For example, should a person become infected with COVID‐19, their mental and physical functioning might be impacted directly, which would reduce their use of nature. Of course, the infection with COVID‐19 is also likely to decrease people's *opportunity* to interact with nature because individuals who are diagnosed with the disease are admitted to hospital or remain home in isolation. More importantly, even if people are not infected with COVID‐19, the uncertainties and fears associated with the virus outbreak, along with mass lockdowns and economic recession, are likely to lead to increased prevalence of symptoms of anxiety, depression, PTSD and other forms of psychological illness in the general population. Indeed, it has been reported that the pandemic is associated with highly significant levels of psychological distress that meet the thresholds for clinical relevance (Ettman et al., 2020; Rajkumar, 2020; Salari et al., 2020; Xiong et al., 2020; Figure 3c). McIntyre and Lee (2020) have reported a projected fivefold increase in the number of suicides associated with joblessness in Canada. These facts suggest that the impact of changes in capability on the dynamics of human–nature interactions could be much greater than generally assumed (Box 1).

This said, the deterioration of mental health conditions due to the pandemic might, in some cases, enhance people's interactions with nature. For example, a recent study conducted in 18 countries has shown that people with common mental health disorders are more likely to visit natural environments than those with no such condition, possibly due to an increased motivation to use these environments for symptom self‐management (Tester‐Jones et al., 2020); there is evidence that people with mild‐to‐moderate mental health disorders may gain the greatest benefits from experiencing nature (Cox et al., 2017). This outcome indicates that changes in *capability* can sometimes lead to changes in *motivation*, highlighting the existence of complex interrelations between the three drivers of human–nature interactions (Box 1).

### 2.3 Pathway 3: Changes in *motivation*

*Motivation* is a person's brain processes that energise and direct behaviour. It is possible that the spread of COVID‐19 disease has altered people's motivation to interact with nature substantially (Table 1; Figure 2). For example, at least in more developed countries, the pandemic has raised many people's motivation to take part in physical exercise, possibly to compensate for reduced everyday physical activity (e.g. travelling to work, physical exertion at work) due to stay‐at‐home orders, a raised awareness of the importance of strengthening immune systems, and ubiquitous messages recommending exercise from media, governments and health authorities (e.g. WHO; Ding et al., 2020). Such increased motivation for physical activity may be a key driver of the widespread significant increase in the use of greenspace (i.e. green exercise; e.g. Derks et al., 2020; Geng et al., 2021; Grima et al., 2020; Kleinschroth & Kowarik, 2020; Venter, Aunan, et al., 2020; Venter, Barton, et al., 2020).

The increased use of natural environments during the pandemic (e.g. Derks et al., 2020; Kleinschroth & Kowarik, 2020; Venter, Aunan, et al., 2020; Venter, Barton, et al., 2020) could be attributed, at least partly, to increases in people's motivation to spend time in nature itself (Grima et al., 2020; Kleinschroth & Kowarik, 2020). This notion stems from the assumption that the pandemic has caused the majority of people to experience higher levels of stress, uncertainty and fear, and, as a result, natural environments might act as a ‘refuge’ in which they can foster psychological stability. In other words, nature can serve as a buffer in decreasing the adverse impacts of major stressful events on human health and well‐being (Corley et al., 2021; Dzhambov et al., 2020; Grima et al., 2020; Pouso et al., 2020; Soga et al., 2021; Theodorou et al., in press). Indeed, especially in more developed countries, during the pandemic, there have been many positive messages in the media about the role of nature experiences in maintaining people's psychological well‐being (e.g. ‘spending time in nature relieves COVID‐stress’, ‘gardening will keep you well during the pandemic’; e.g. BBC News 3/5/2020, Guardian 20/5/2020). Also, there is some evidence that with more people working from home, and therefore theoretically free to work from anywhere with internet access, there has been increased interest in moving from large cities to live in more rural locations with larger gardens and better access to nature (e.g. BBC News 18/11/2020, Japan Times 4/11/2020). These facts raise the possibility that the extent of the health and well‐being benefits derived from nature interactions, as well as societal awareness of these benefits, might have increased during the pandemic (Grima et al., 2020; Figure 3d), although further studies are needed to confirm this idea (Box 1).

There is a growing body of scientific and anecdotal evidence showing that, at least in more developed societies, people's positive attitudes towards nature (especially local wildlife and natural environments) have increased during the pandemic. For example, a large questionnaire survey showed that 74% of adults in England agreed that they had noticed more nature in their neighbourhoods since the onset of the pandemic than they would normally at that time of year (RSPB, 2020). Likewise, Rousseau and Deschacht (2020) found that public interest in nature (measured by people's internet search behaviour) increased substantially in 20 European countries during the pandemic (Figure 3e). We suspect, however, that the increased positive attitudes towards nature observed at this time have not directly been caused by the pandemic itself. Rather, we consider that the increased positive attitudes were secondarily induced by increased frequencies of nature interactions that have been caused by other factors (e.g. increased availability of discretionary time, raised awareness of health; see Section 2.4). Nevertheless, this can be seen as a positive because increased interest in, and emotional affinity towards, nature is likely to promote people's engagement with it, as this is found to be a key driver of human–nature interactions (e.g. Lin et al., 2014; Soga et al., 2018; Soga & Akasaka, 2019).

The pandemic could also decrease some people's motivation to interact with nature. For example, it is increasingly apparent that it has led to substantial growth in negative public attitudes towards bats (known to be vectors or reservoirs for many strains of coronavirus; Cerri et al., 2020). Increased negative feelings and attitudes towards wildlife, often called ‘biophobia’ (Soga et al., 2020; Zhang et al., 2014), may reduce people's willingness to engage with ‘wild’ nature (Box 1). This situation would be somewhat similar to the period of dengue epidemics in Tokyo in 2014, when increased negative attitudes towards mosquitoes (vectors of dengue virus) caused people to temporary avoid using local greenspaces (M.S., pers. obs.).

*Motivation* is often associated with *opportunity*, as the former can drive the latter (Figure 2). For example, increased interest in nature during the pandemic (i.e. changes in *motivation*) might enhance people's motivation for participating in wildlife gardening in their domestic gardens, which is likely to boost the abundance and species richness of wildlife around their home (i.e. changes in *opportunity*; Cox & Gaston, 2018). On the other hand, an increase in people's negative attitudes towards bats (e.g. concerns over the risk of exposure to the virus) has the potential to decrease the abundance and distribution of these organisms (Cerri et al., 2020; MacFarlane & Rocha, 2020; Sasse & Gramza, 2021). Understanding the relationships among the three drivers of changes in human–nature interactions due to the pandemic is a key challenge (Box 1).

### 2.4 Feedback loops

The changes in human–nature interactions that have been induced by the pandemic could result in further alteration of the dynamics of these interactions (Figure 2). For example, in an urban context, increased visits to greenspace caused by the pandemic could promote people's physical and mental health (e.g. Pouso et al., 2020; Soga et al., 2021; Table 1), as well as their emotional affinity towards nature (Grima et al., 2020), which may, in turn, increase their *capability* and *motivation* to interact with nature (Figure 2). Likewise, an increase in people's use of nearby (e.g. protected areas) and neighbourhood (e.g. urban greenspace) natural environments can result in increased habituation of wildlife to humans (e.g. decreased escape responses; Uchida et al., 2019), which may increase their *opportunity* to interact with wildlife more directly (Figure 2). These three types of pathways imply that there exists a ‘positive’ feedback process by which increases in human–nature interactions can result in further increases in these interactions. This raises a possibility that the pandemic may have an important influence on the ongoing, widespread loss of human–nature interactions (the ‘extinction of experience’, Miller, 2005; Soga & Gaston, 2016), although more empirical research, particularly from long‐term studies, is required to test this idea (Box 1).

Of course, there may also be negative feedback loops where increases (or decreases) in human–nature interactions lead to decreases (or increases) in these interactions. In the case of greenspace and protected area use, for example, there have been many anecdotal observations of erosion and loss of understorey vegetation due to increased use of those environments by local people (e.g. jogging, walking, hiking, dog walking) during the pandemic, which is likely to decrease the species richness and abundance of wildlife species (e.g. birds, butterflies) people can interact with (i.e. loss of *opportunity*). Also, some human‐sensitive species (e.g. ground‐dwelling birds) will become less abundant in response to the presence of a large number of greenspace or protected area visitors (Bötsch et al., 2018; Lethlean et al., 2017; Thompson, 2015). More importantly, the overuse of greenspaces and protected areas during the pandemic might reduce the environmental quality of these environments (e.g. increased litter, increased noise levels, more frequent fires), which is also likely to decrease people's willingness to use these environments (i.e. loss of *motivation*; of course, the presence of large numbers of other people itself can also decrease the motivation of some individuals to use these environments). Understanding how the increased visitations to natural environments during the pandemic can affect people's *opportunity* and *motivation* to use these environments can provide fundamental insights into how sustainably to manage natural environments at times of societal stress.

## 3 CONCLUSION

The dramatic changes in people's lifestyles and social systems associated with the pandemic have led to an unprecedented alteration of the dynamics of human–nature interactions worldwide. Although it is uncertain how long this situation will continue, the impacts of the pandemic on human–nature interactions seem to be likely to last for years, including after it has ended (as a legacy effect; Box 1). Indeed, the pandemic has brought about changes in the lifestyle, norms and attitudes of people in many ways, some of which will remain over the longer term (e.g. the adoption of remote working practices, the establishment of regular exercise habits in urban parks, increased awareness of the importance of nature experiences). Likewise, the feedback loops within our framework suggest that the pandemic could have time‐lagged or cumulative effects on human–nature interactions (Box 1). Given these potentially long‐term and widespread consequences of the pandemic, researchers can use this extraordinary period as a ‘global natural experiment’ (Thomson, 2020) to gain novel insights into the complex processes and dynamics of these interactions and into possible strategies to manage them to best effect (see Box 1 for a list of priority research questions). Indeed, as discussed throughout this paper, the knowledge gained from such an approach could have the potential to inform the development of policies and strategies to address some of the most significant challenges related to human–nature interactions, such as minimising negative consequences of the health‐associated demands on greenspace (Stanley et al., 2015), preventing the ongoing, widespread loss of positive human–nature interactions (Soga & Gaston, 2016), and mitigating human–wildlife conflicts in rural and suburban areas (Tsunoda & Enari, 2020). To take maximum advantage of this window of opportunity, therefore, we recommend that researchers, alongside policy‐makers and practitioners (e.g., city planners, protected area managers and health professionals) establish testable hypotheses and, where possible, collect data sooner rather than later. Although undeniably tragic, the pandemic may offer an invaluable opportunity to explore an appropriate future relationship between people and nature far beyond that concerned with animals as sources of novel human coronavirus infections.

## CONFLICT OF INTEREST

K.J.G. is an Editor‐in‐Chief of *People and Nature*, but took no part in the peer review and decision‐making processes for this paper.

## AUTHORS' CONTRIBUTIONS

M.S. and K.J.G. conceived the ideas and designed the conceptual framework; M.S. led the writing of the manuscript. All authors contributed critically to the drafts and gave final approval for publication.

## Supporting information

Supplementary Material

## Data Availability

No data were collected for this paper.
