# Supplementary material for: Impacts of the COVID‐19 pandemic on human–nature interactions: Pathways, evidence and implications
Source: People Nat (Hoboken). 2021 Apr 6;3(3):518–27. doi: 10.1002/pan3.10201 (PMC8251160; doi:10.1002/pan3.10201)
Supplement: Supplementary file 1 — Supplementary Material [file PAN3-3-518-s001.pdf]

PEOPLE AND NATURE

How has the COVID-19 pandemic affected human-nature interactions?

Masashi Soga, Maldwyn J. Evans, Daniel T.C. Cox, Kevin J. Gaston

The COVID-19 pandemic has dramatically changed people's lifestyles in much of the world. These major changes can alter the dynamics of the direct interactions between humans and nature (hereafter human-nature interactions) far beyond those concerned with animals as sources of novel human coronavirus infections. For example, there is considerable evidence that, in many urbanised societies, people's use of neighbourhood nature soared during the pandemic. It has also been reported that during the pandemic, due to reduced human movement, there have been fewer collisions between vehicles and wildlife on highways. These widespread changes in human-nature interactions can have a variety of consequences for both people and nature.

In this review, we suggest a conceptual framework for understanding how the pandemic might affect the dynamics of human-nature interactions and the consequences thereof. Our framework illustrates that the pandemic can affect human-nature interactions through three different pathways: changes in (i) opportunity (e.g., the amount of wildlife, the amount of time available for nature-based activities), (ii) capability (e.g. mental and physical functioning) and (iii) motivation (e.g. inclination towards engaging with nature). Through this framework, we also suggest that the impacts of the pandemic on human-nature interactions seem to be likely to last for years, including after it has ended.

Our review suggests that, although undeniably tragic, the pandemic may offer an invaluable opportunity to gain novel insights into the complex dynamics of human-nature interactions and into possible strategies to manage them to best effect. Scientific knowledge during this time could have the potential to inform the development of policies and strategies to address some of the most significant challenges related to human-nature interactions, such as minimising negative consequences of the health-associated demands on greenspace, preventing the ongoing, widespread loss of positive human-nature interactions and mitigating human-wildlife conflicts in rural and suburban areas.

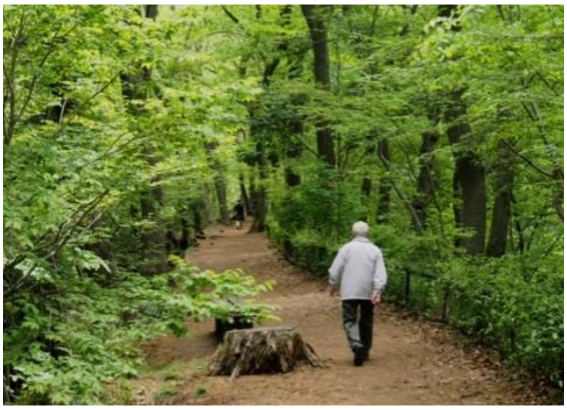

In many urbanised societies, people's use of urban greenspaces has increased substantially during the COVID-19 pandemic (photo credit: Masashi Soga).
